# Fluidity and Inconstancy: Australian Bush Tomatoes as an Exemplar of Non-Normative Sex Expression

**DOI:** 10.1093/icb/icad051

**Published:** 2023-06-01

**Authors:** Christopher T Martine

**Affiliations:** Department of Biology, Bucknell University, Lewisburg, PA 17837, USA

*This has me puzzled, also. It is obviously one of the andromonoecious species, but lacks the male racemes above the fertile flower. I have not seen its like before, but I think it some variant or aberrant*.

- David Symon, August 1976, note on Peter Latz collection 5482

The above annotation on a 1974 collection of an unusual Australian bush tomato (*Solanum*) made by Peter Latz says a lot more than it may at first seem. This note, on a herbarium sheet in the Northern Territory Herbarium in Palmerston (Fig. 1), represents a tellingly frank admission of puzzlement from the late David Symon—not only Australia’s foremost nightshade (plant family Solanaceae to which *Solanum* belongs) expert in history but the eventual author of the continent’s only monograph of the genus *Solanum* (Symon 1981), a book that, among its many pages, includes dozens of species described and named by Symon himself. That Symon, who spent decades studying floral variation among species in this group, would be stumped by what he saw on this particular specimen is an indication of something quite unexpected. It was certainly enough for that specimen to be filed away in a cabinet with the other “indetermined” collections, where it would remain for the next 40+ years.

**Fig. 1:**
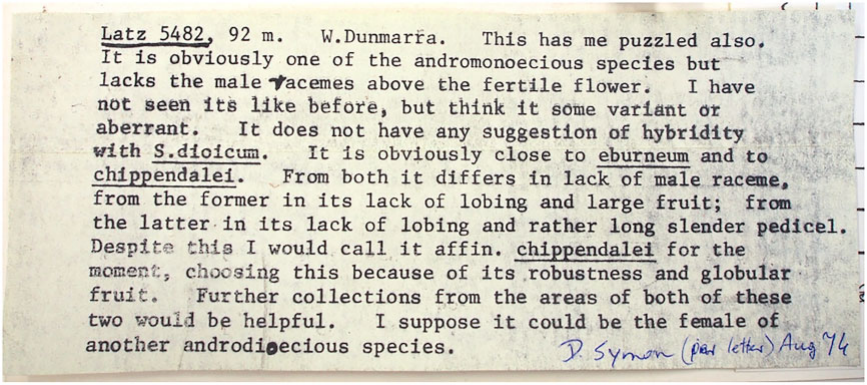
Annotation label with comments from David Symon, attached to Peter Latz collection 5482 held at the Northern Territory Herbarium (Palmerston). Photo by C.T. Martine.

What was it that Symon expected to see? What would have allowed him to feel confident about the sexual identity of this particular pressed bit of vegetation? At that time, there were understood to be three possible options for sexual system expression among the bush tomato species of the Australian Monsoon Tropics (a clade of taxa with close affinity to the cultivated eggplant):

1. Cosexuality (here used in place of what many previous authors have called “hermaphroditism”), in which all flowers on all plants bear both “male” (staminate) and “female” (carpellate) reproductive organs; and in which both pollen and fruit/seed production are possible in every flower.
2. Andromonoecy, in which every individual plant bears flower clusters consisting of a (typically) single cosexual flower plus numerous unisexually “male” flowers; where every flower produces functional pollen but only the cosexual flowers can set fruit/seeds. Symon likely assumed that the exasperating specimen above was “obviously” andromonoecious because of the position of the flowers, the length of their stalks, and the spaces along the stems where “male” flowers ought to be.
3. Androdioecy (which would later prove to be a misnomer given the discovery of functional dioecy in *Solanum* described below), in which some plants bear only solitary cosexual flowers (with potential for fruit/seed production) and other plants bear only clusters of unisexually “male” flowers.

The Latz collection’s apparent unwillingness to be placed neatly into one of these discrete bins was enough for it to be declared “aberrant” by Symon, but the seemingly fluid nature of this collection would turn out to be less of an aberration than a harbinger of scientific findings to come.

Only a few years after Symon’s annotation, Greg Anderson would discover that another reproductively odd *Solanum* species (from Central America) would turn out to be much more than met the eye: The morphologically cosexual flowers borne by some plants did have both stamens (“male” parts) and pistils (“female” parts), but the stamens were found to produce non-functional pollen (Anderson 1979). Whereas regular *Solanum* pollen has pores in the pollen wall to allow the germination of a pollen tube and delivery of sperm cells following its growth through a stigma and down a style, these pollen grains had none (they were without pores, or inaperturate). In other words, while these flowers appear to be cosexual, they are strictly “female” in the functional sense. Meanwhile, the flowers borne by a third variant of this same species (recognized as a separate species at the time!) were also cosexual, that is, they also had “male” and “female” parts. In these flowers, the “female” organs were much reduced, and never produced fruit (even when hand pollinated with pollen from separate plants). Their anthers, however, bore the typical three-pored pollen that typifies these plants (and potatoes and tomatoes), so these plants were functionally “male”—and thus the functional sex system is best described as dioecy, a system in which unisexual “male” and “female” flowers occur on separate plants. Dioecy is widespread (see *Cannabis*, willows, and hollies for common examples) but still considered relatively rare among flowering plants (Renner 2014).

Anderson and Symon (1989) would later apply the lessons learned through the above Central American species to an extensive reproductive biology comparison across a set of andromonoecious and “androdioecious” Australian bush tomatoes, finding that the latter were indeed also best described as functionally dioecious, characterized by the inaperturate pollen of the “female” flowers. In these taxa, functionally female flowers/plants “masquerade” as cosexual (Fig. 2)—likely because the dependence on pollen-foraging bees for pollination (there is no nectar reward in *Solanum*; see Anderson and Symon 1988; Ndem-Galbert et al. 2021) precludes any loss of a pollen reward and the organs that produce and present it even in the face of assumed selective pressures to be obligately outcrossing (see Cantley et al. 2023). Every Australian bush tomato species previously identified as androdioecious has now been confirmed as functionally dioecious, plus a number of species described since Symon’s monograph that share the same morphology (e.g., Brennan et al. 2006; Martine et al. 2016; Williams et al. 2022). The andromonoecious group of taxa, to which Symon’s “aberration” seemed to belong, has long been inferred to represent a transitional sex system on the path to a (perhaps problematically teleological) “final destination” of functional dioecy (see Martine and Anderson 2007).

**Fig. 2:**
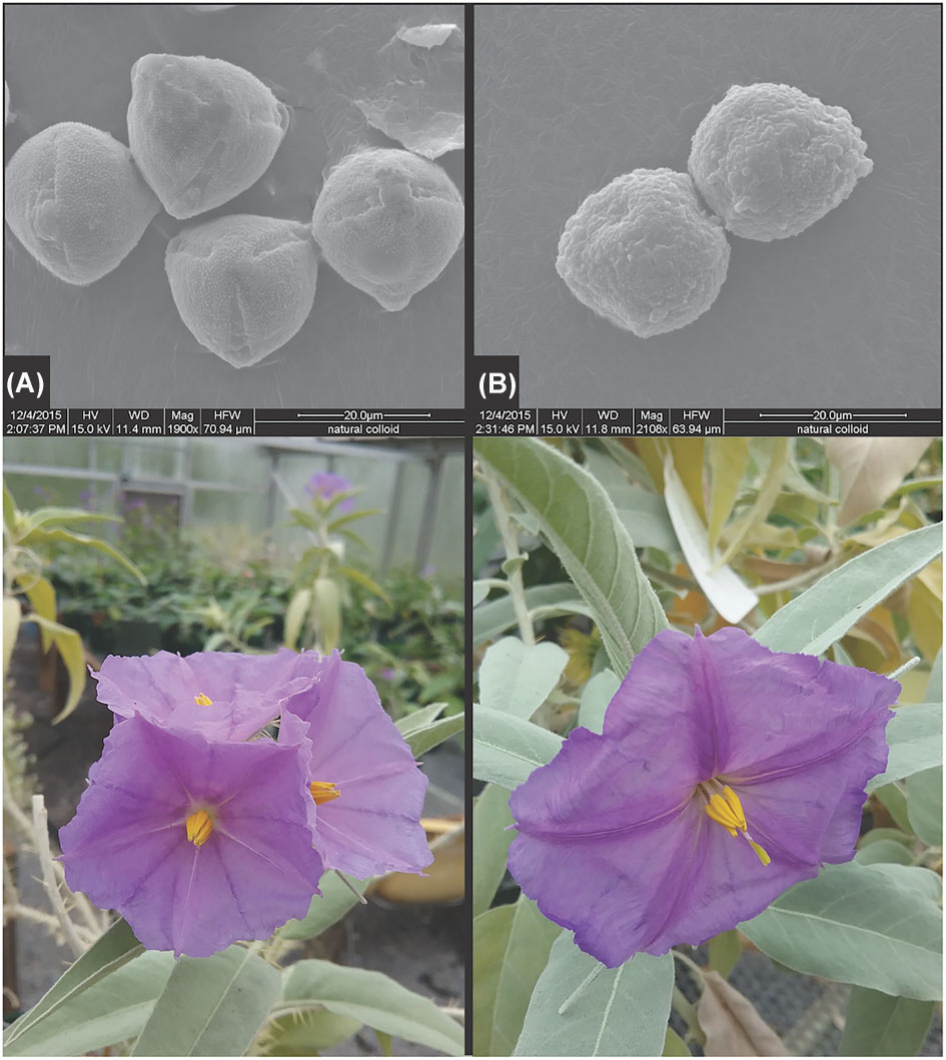
Flower and pollen morphology of *Solanum ossicruentum*, a functionally dioecious bush tomato from Australia. Column (A) functional porate pollen (above) produced by “male” flowers/plants; column (B) non-functional inaperturate pollen (above) produced by morphologically cosexual flowers/plants, rendering them functionally “female.” Photo plate published previously in Martine et al. (2016).

However, recent phylogenetic work by my colleagues and I (Martine et al. 2019; McDonnell and Martine in prep) suggests that dioecy is not an end point in Australian *Solanum*. Instead, our results point to a reversal from dioecy back to cosexuality (the ancestral state in the genus), an inference that runs counter to suggestions that dioecy in flowering plants tends to be an evolutionary dead end (see Vamosi and Otto 2002). Despite the perceived unlikelihood of this reversal, evidence from field and greenhouse observations points to a probable pathway: leaky dioecy.

Coined by Baker and Cox (1984), leaky dioecy is a form of sex inconstancy in which typically unisexual flowers sometimes develop the organs and function of the opposite sex. In dioecious *Solanum*, this occasionally occurs in “male” flowers that, in addition to producing pollen, also form a fully functional gynoecium (the sum of all “female” parts)—and might then be called “leaky males” (Fig. 3). We have now seen leaky males, capable of setting fruit, in at least six functionally dioecious Australian bush tomato species. While the fruits set on these “male” plants are smaller than fruits produced on “female” plants and contain far fewer seeds (in one species that typically produces ∼400 seeds per fruit, “male fruits” contain ∼10–30), those seeds are often viable. Incredibly, seeds grown from leaky male bush tomato fruits result in 100% recruitment of “male” individuals in the next generation (Hayes 2018)—a phenomenon first reported for the Caribbean dioecious species *Solanum polygamum* by Anderson et al. (2015)—rather than the more-or-less 50:50 “male/female” ratio seen over our ∼20 years of field counts and greenhouse recruitment observations. These new all-male progeny grow up to be leaky males themselves, essentially creating a swarm of individuals who are now both morphologically and functionally cosexual. As such, a population of dioecious bush tomatoes might transition to (or establish via seed dispersal) a population that is cosexual—thus providing us with a putative pathway for the sex system reversal we are seeing in our phylogenies. This is aided by the fact that all “male” *Solanum* flowers still contain a vestigial gynoecium nestled deeply beneath the stamens, almost always too tiny and undeveloped to be functional—but, as in the case of leaky dioecy, with a rare capacity to develop to maturity.

**Fig. 3:**
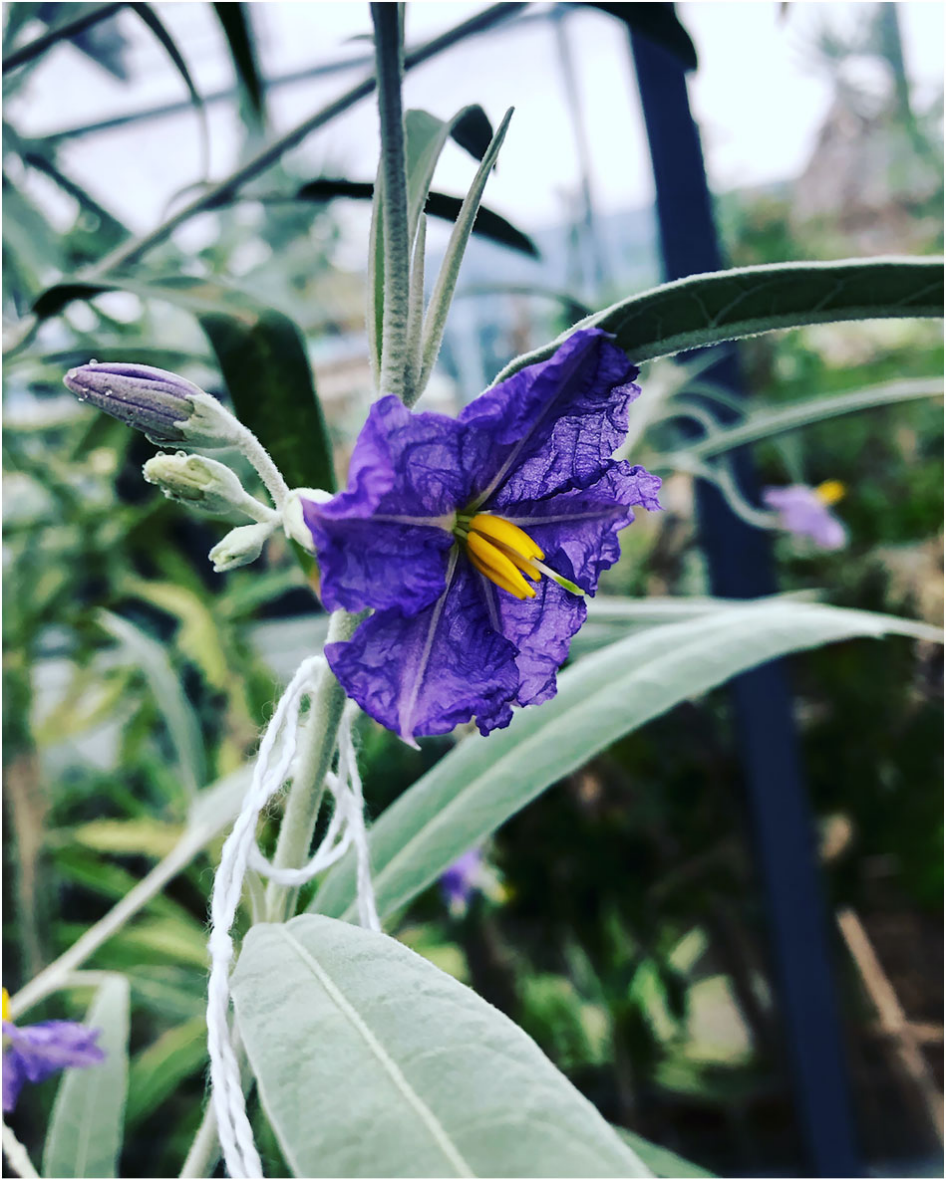
Leaky “male” flower on a functionally dioecious *Solanum tudununggae* plant in cultivation at Bucknell University. Note the fully exerted style and green stigma. Photo by C.T. Martine.

Symon’s confusion over the “aberrant” collection and its failure to be easily placed in an established sexual system was not born from an anomaly. Instead, fluidity and inconstancy in sex expression appear to be the normative conditions among Australia’s bush tomatoes. Dioecy is not always really dioecy; sometimes “males” are cosexuals. Likewise, some andromonoecious bush tomatoes have been recorded as showing “weak andromonoecy” (e.g., Bean 2012) to describe instances when cosexual flowers (typically appearing as a single flower at the base of the cluster) occasionally occur in distal “male” positions. The categories we employ to describe how these plants express their sexuality, while useful, are not always discreet and non-overlapping; there is often a gray area in-between—and that is where the most exciting observations might be made.

When my colleagues and I came across the very same population of “aberrant” plants while working in the field in 2018, we were also confused by what we saw. Some plants only bore male flowers, others only cosexual flowers, and still others presented as andromonoecious. When we later encountered the 1974 specimen mentioned above in its “indetermined” folder, we realized that not only was this an undescribed species with a previously unencountered level of sexual fluidity but that this inconstant condition provided an excellent example of the variation in sex expression seen across the plant kingdom. To honor this variation, we named the species *Solanum plastisexum* and concluded the paper describing it (McDonnell et al. 2019) with the following broad (and likely obvious to many biologists) statement on both plants and non-plants:

*The notion of a constant sexual binary consisting of two distinct and disconnected forms is fundamentally a fallacy*.

Diversity in sexual expression is certainly the rule among plants, not the exception. I am only a botanist, but I do not believe it is a reach to suggest that this also holds true for the rest of life on Earth. Some might see the quoted passage above as a sort of “straw man” that has already been thoroughly refuted, but one does not need to look very far to see that arguments for binary sexuality as the rule are persistent. To wit: the social media response to a recent Wall Street Journal op-ed by an evolutionary biologist (Wright 2023) arguing for “binary sex” and criticizing “gender ideologues” manifested as more than a million impressions and thousands of comments within days of publication.

Still, is extrapolating what is happening in a clade of Australian bush tomatoes to humans appropriate? Can/should we apply the lessons learned through the close study of sexual fluidity in *Solanum* to the study of other organismal groups? At minimum, it's a case study in how assumptions regarding sexual expression can hinder understanding of the organisms/systems we work on—and, perhaps, this is another in a growing list of opportunities to recognize similar biases in how we approach our work on all organisms, humans included.

## Notes

From the symposium “Sex Diversity and Variation” presented at the annual meeting of the Society for Integrative and Comparative Biology, January, 2023 at Austin, Texas.
